# Efficacy and safety of transjugular intrahepatic portosystemic shunt in hepatic sinusoidal obstruction syndrome: systematic review and meta-analysis

**DOI:** 10.3389/fmed.2025.1625825

**Published:** 2025-08-13

**Authors:** Xinghe Jiang, Xuan Ma, Shuni Tian, Lijun Peng

**Affiliations:** ^1^School of Clinical Medicine, Shandong Second Medical University, Weifang, Shandong, China; ^2^Department of Gastroenterology, Linyi People’s Hospital Affiliated to Shandong Second Medical University, Linyi, Shandong, China; ^3^Department of Gastroenterology, Peking University People’s Hospital Qingdao, Qingdao, Shandong, China

**Keywords:** hepatic sinusoidal obstruction syndrome, hepatic veno-occlusive disease, transjugular intrahepatic portosystemic shunt, portal hypertension, meta-analysis

## Abstract

**Background:**

Hepatic sinusoidal obstruction syndrome (HSOS) is a rare but life-threatening liver condition. The clinical utility of transjugular intrahepatic portosystemic shunt (TIPS) for HSOS remains unclear.

**Methods:**

A systematic review and meta-analysis was performed across five databases (up to November 2024) to evaluate the efficacy and safety of TIPS in HSOS patients. Key outcomes included technical success, clinical response, portal pressure changes, survival, and complications.

**Results:**

Nineteen studies involving 465 patients were included. Pooled technical success and clinical response rates were 100 and 94.2%, respectively. TIPS significantly reduced portal pressure (mean PPG: −13.5 mmHg; PVP: −12.3 mmHg). The 3-month and 1-year survival rates were both 91.6%. Hepatic encephalopathy occurred in 13.2% of patients.

**Conclusion:**

TIPS is a feasible and relatively safe treatment for HSOS, particularly in patients with pyrrolizidine alkaloid–induced HSOS (PA-HSOS). Further research is warranted to confirm its long-term benefits.

**Systematic review registration:**

https://www.crd.york.ac.uk/PROSPERO/view/CRD42024620323, identifier CRD42024620323.

## Introduction

Hepatic sinusoidal obstruction syndrome (HSOS), formerly known as hepatic veno-occlusive disease (HVOD), is a hepatic vascular disorder characterized by sinusoidal endothelial cell injury, leading to edema, necrosis, endothelial cell detachment, and microthrombosis within the hepatic sinusoids, central venules, and interlobular veins (1). These hemodynamic alterations can lead to portal hypertension and impaired liver function, potentially progressing to multi-organ failure and death. In developed countries, HSOS is most commonly observed in patients undergoing hematopoietic stem cell transplantation (HSCT) following myeloablative conditioning, those receiving chemotherapy for solid malignancies, or liver transplant recipients on immunosuppressive therapy. However, in contrast to developed countries, HSOS in China is primarily attributed to the consumption of pyrrolizidine alkaloid (PA)-containing herbs, such as *Gynura segetum*, due to their widespread use in traditional medicine (2–4).

Although there are a large number of HSCT cases in China, HVOD is rarely reported in HSCT patients, likely due to insufficient awareness of the condition. Due to potential differences in ethnicity, etiology, and underlying diseases, there is no global consensus on the diagnosis and treatment of HSOS. At present, the treatment of HSOS mainly focuses on anticoagulation therapy and symptomatic supportive measures, including liver protection, diuretics, and improving microcirculation (5). In critically ill patients, the mortality rate remains high with supportive treatment alone (6). Therefore, the prognosis for patients with severe HSOS remains poor, and new strategies are needed to improve their outcomes. TIPS is an effective interventional procedure. This procedure alleviates hepatic congestion by shunting portal venous blood flow, thereby reducing portal pressure and improving liver function, with an extremely low procedure-related mortality rate (7, 8). Studies have shown that TIPS can alleviate portal hypertension and improve liver function in HSOS patients, providing additional time for liver transplantation (9).

Nevertheless, due to the absence of sufficiently robust randomized controlled trials, the efficacy and safety of TIPS in HSOS patients remain inconclusive. To address this knowledge gap, the present study conducts a meta-analysis of existing published studies to systematically evaluate the efficacy and safety of TIPS in the management of HSOS.

## Methods

This systematic review and meta-analysis of single-arm studies was conducted in strict accordance with the Preferred Reporting Items for Systematic Reviews and Meta-Analyses (PRISMA) guidelines (10).

### Literature search

Five databases, including PubMed, Embase, Scopus, Web of Science and the Cochrane Library, were searched to identify eligible studies. The search period covered all records from database inception to November 30, 2024. The search keywords were: “Portasystemic Shunt, Transjugular Intrahepatic; Shunt, Transjugular Intrahepatic Portasystemic; TIPSS; Shunt, Transjugular Intrahepatic Portosystemic; Portosystemic Shunt, Transjugular Intrahepatic; TIPS; Transjugular Intrahepatic Portasystemic Shunt; Hepatic Veno-Occlusive Disease; Disease, Hepatic Veno-Occlusive; Hepatic Veno-Occlusive Diseases; Sinusoidal Obstruction Syndrome; Syndrome, Sinusoidal Obstruction; Hepatic Veno Occlusive Disease; Veno-Occlusive Disease, Hepatic; Veno Occlusive Disease, Hepatic” For example, the search strategy in PubMed was as follows: ((“Portasystemic Shunt, Transjugular Intrahepatic”[Mesh]) OR (((((((Portasystemic Shunt, Transjugular Intrahepatic[Title/Abstract]) OR (Shunt, Transjugular Intrahepatic Portasystemic[Title/Abstract])) OR (TIPSS[Title/Abstract])) OR (Shunt, Transjugular Intrahepatic Portosystemic[Title/Abstract])) OR (Portosystemic Shunt, Transjugular Intrahepatic[Title/Abstract])) OR (TIPS[Title/Abstract])) OR (Transjugular Intrahepatic Portasystemic Shunt[Title/Abstract]))) AND ((“Hepatic Veno-Occlusive Disease”[Mesh]) OR ((((((((Hepatic Veno-Occlusive Disease[Title/Abstract]) OR (Disease, Hepatic Veno-Occlusive[Title/Abstract])) OR (Hepatic Veno-Occlusive Diseases[Title/Abstract])) OR (Sinusoidal Obstruction Syndrome[Title/Abstract])) OR (Syndrome, Sinusoidal Obstruction[Title/Abstract])) OR (Hepatic Veno Occlusive Disease[Title/Abstract])) OR (Veno-Occlusive Disease, Hepatic[Title/Abstract])) OR (Veno Occlusive Disease, Hepatic[Title/Abstract]))). No language restrictions were applied during the search. Additionally, gray literature and the references of the included articles were also searched to ensure that no eligible studies were overlooked.

### Study selection

All studies were independently evaluated by two authors, with any disagreements resolved through discussion with a third author. The selection process involved searching the literature using predefined keywords, screening study titles and abstracts, and reviewing the full text of potentially eligible studies. Reasons for exclusion and the number of excluded studies were documented. The inclusion criteria were as follows: (1) Population: Patients diagnosed with HSOS, (2) Intervention: Treatment with TIPS, (3) Outcomes: Efficacy and safety of TIPS in HSOS. The exclusion criteria were: (1) Pediatric patients (age < 10 years), (2) Studies lacking relevant outcome measures or with incomplete data, (3) Conference abstracts, case reports, review articles, letters, and editorials, (4) Duplicate publications.

### Data extraction

Data extraction was independently performed by two authors, with a third author assessing the clinical outcomes. Discrepancies were resolved through discussion and consensus. Key data extracted from the original studies included both general information and clinical outcomes. General information encompassed the first author’s name, publication year, country, etiology, study design, interventions, sample size, patient age, and follow-up duration. Clinical outcomes were divided into two categories: safety and efficacy. Efficacy endpoints included the technical success rate, clinical response rate, and mean changes in portal pressure gradient (PPG) and portal vein pressure (PVP). Safety endpoints included the 3-month survival rate, 1-year survival rate, and the incidence of HE.

### Risk assessment

Given the single-arm design of this study without a control group, the Joanna Briggs Institute (JBI) Critical Appraisal Checklist for Case Series was used to assess the risk of bias (11). To ensure consistency, all included studies were independently evaluated, and any disagreements were resolved through consensus.

### Statistical analysis

Data were collected and standardized from various reports, and a single-arm meta-analysis was performed using Stata version 16.0 to evaluate the efficacy and safety of TIPS in the treatment of HSOS. Heterogeneity among studies was assessed using Cochrane’s Q chi-squared test and the I^2^ statistic. For low to moderate heterogeneity (*p* ≥ 0.10 and *I*^2^ < 50%), a fixed-effects model was applied, whereas a random-effects model was used for high heterogeneity (*p* < 0.10 or *I*^2^ > 50%). To address high heterogeneity in the pooled results, sensitivity analyses were conducted by excluding studies one at a time. Publication bias was evaluated using funnel plots, with Egger’s and Begg’s tests. Additionally, subgroup analyses were performed to explore the influence of different etiologies on the efficacy and safety of TIPS in the treatment of HSOS.

## Results

### Study selection and characteristics

**FIGURE 1:**
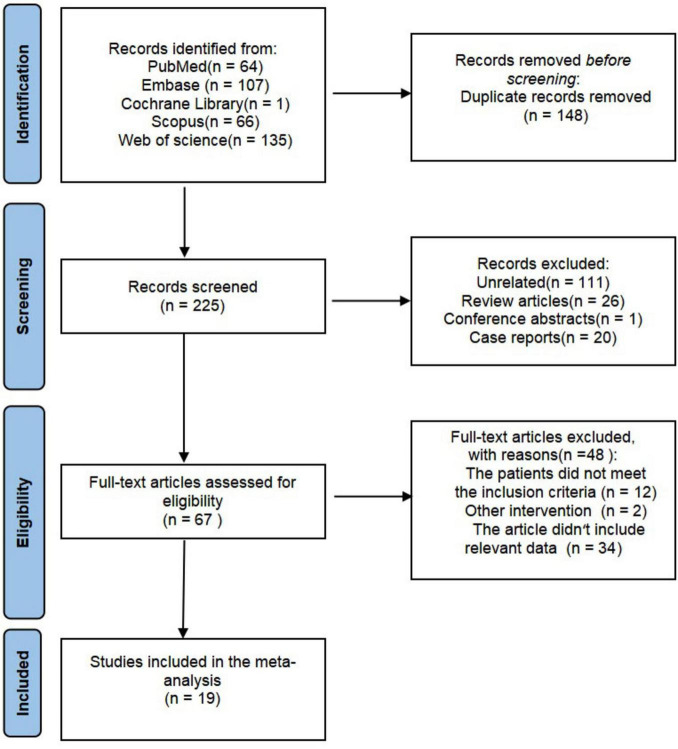
Flow diagram of the selection process.

Our initial search across five databases identified a total of 373 articles, comprising 107 from Embase, 64 from PubMed, 135 from Web of Science, 1 from the Cochrane Library, and 66 from Scopus. After removing duplicate studies, screening the titles and abstracts of the remaining records, and reviewing the full text of potentially eligible studies, 19 studies involving a total of 465 patients met the inclusion criteria and were included in this meta-analysis (12–30). All included studies were retrospective in design. The study selection process is illustrated in Figure 1, and the details of each included study are provided in Table 1. The review protocol was registered with the International Prospective Register of Systematic Reviews (PROSPERO) under registration number CRD42024620323.

**TABLE 1:** Characteristics of the included studies.

Study	Country	Study design	Etiology	Patients, n	Gender	Age (years)	Follow-up (months)	AT before TIPS
Gómez-Centurión et al. (12)	Spain	Retrospective	HSCT-HSOS	7	All Male	22–36	4–84	6Y, 1N
Zhou et al. (13)	China	Retrospective	PA-HSOS	37	28M, 9F	62.3 (Mean)	3–48	YES
Zhang et al. (14)	China	Retrospective	PA-HSOS	9	6F, 3F	49–78	5–39	7Y, 2N
Huang et al. (15)	China	Retrospective	PA-HSOS	12	7M, 5F	61.5 (Mean)	10.5 (Median)	YES
Wang et al. (16)	China	Retrospective	PA-HSOS	69	39M, 30F	52.2 (Mean)	38.5 (Median)	NO
Li et al. (17)	China	Retrospective	PA-HSOS	13	10M, 3F	49.5–66	NA	NO
Wu et al. (18)	China	Retrospective	PA-HSOS	4	All Female	48–61	NA	YES
Zhuge et al. (19)	China	Retrospective	PA-HSOS	29	NA	NA	NA	27Y, 2N
Huang (20)	China	Retrospective	PA-HSOS	20	11M, 9F	64.1 (Mean)	2–69	NO
Azoulay et al. (21)	France	Retrospective	HSCT-HSOS	10	8M, 2F	11–52	NA	NA
Fried et al. (22)	America	Retrospective	HSCT-HSOS	6	4M, 2F	42 (Mean)	NA	YES
Wang et al. (23)	China	Retrospective	PA-HSOS	27	16M, 11F	21–60	NA	NO
Wang et al. (24)	China	Retrospective	PA-HSOS	7	4M, 3F	29–74	NA	YES
Changlong et al. (25)	China	Retrospective	PA-HSOS	21	16M, 5F	25–86	NA	YES
Wei et al. (26)	China	Retrospective	PA-HSOS	9	5M, 4F	53–75	1–17	NO
Yi et al. (27)	China	Retrospective	PA-HSOS	21	NA	NA	NA	YES
Xiao et al. (28)	China	Retrospective	PA-HSOS	116	72M, 44F	63.6 (Mean)	NA	86Y, 30N
Xu et al. (30)	China	Retrospective	PA-HSOS	15	7M, 8F	30–85	NA	NO
Zhang et al. (29)	China	Retrospective	PA-HSOS	54	NA	NA	NA	YES

PA-HSOS, pyrrolizidine alkaloid-induced hepatic sinusoidal obstruction syndrome; HSCT-HSOS, hematopoietic stem cell transplantation-induced hepatic sinusoidal obstruction syndrome; AT, anticoagulant treatment; TIPS, transjugular intrahepatic portosystemic shunt; NA, not available.

### Quality assessment

All studies were evaluated using the JBI Critical Appraisal Checklist for Case Series. Detailed results of the assessment are presented in Table 2.

**TABLE 2:** Quality assessment of individual studies according to the JBI critical appraisal checklist.

Study	Q1	Q2	Q3	Q4	Q5	Q6	Q7	Q8	Q9	Q10	Overall appraisal
Gómez-Centurión et al. (12)	YES	YES	YES	YES	YES	YES	YES	YES	YES	YES	Included
Zhou et al. (13)	YES	YES	YES	YES	YES	YES	YES	YES	YES	YES	Included
Zhang et al. (14)	YES	YES	YES	YES	YES	YES	YES	YES	YES	YES	Included
Huang et al. (15)	YES	YES	YES	YES	YES	YES	YES	YES	YES	YES	Included
Wang et al. (16)	YES	YES	YES	YES	YES	YES	Unclear	YES	YES	YES	Included
Li et al. (17)	YES	YES	YES	YES	YES	YES	YES	YES	YES	YES	Included
Wu et al. (18)	YES	YES	YES	YES	YES	YES	YES	YES	YES	Unclear	Included
Zhuge et al. (19)	YES	YES	YES	YES	YES	YES	YES	Unclear	YES	YES	Included
Huang et al. (20)	YES	YES	YES	YES	YES	YES	YES	Unclear	YES	YES	Included
Azoulay et al. (21)	YES	YES	YES	Unclear	YES	YES	Unclear	YES	YES	Unclear	Included
Fried et al. (22)	YES	YES	YES	YES	YES	YES	YES	YES	YES	Unclear	Included
Wang et al. (23)	YES	YES	YES	YES	YES	YES	YES	YES	YES	YES	Included
Wang et al. (24)	YES	YES	YES	YES	YES	YES	YES	YES	YES	YES	Included
Changlong et al. (25)	YES	YES	YES	YES	YES	YES	YES	Unclear	YES	YES	Included
Wei et al. (26)	YES	YES	YES	YES	YES	YES	YES	YES	YES	YES	Included
Yi et al. (27)	YES	YES	YES	YES	YES	YES	YES	YES	YES	YES	Included
Xiao et al. (28)	YES	YES	YES	Unclear	YES	YES	Unclear	YES	YES	YES	Included
Xu et al. (30)	YES	YES	YES	YES	YES	YES	YES	YES	YES	YES	Included
Zhang et al. (29)	YES	YES	YES	YES	YES	YES	YES	YES	YES	Unclear	Included

JBI: Joanna Briggs Institute; Possible answers: Yes, no, unclear, not applicable. No means high risk of bias; Q1: Were there clear criteria for inclusion in the case series? Q2: Was the condition measured in a standard, reliable way for all participants included in the case series? Q3: Were valid methods used for identification of the condition for all participants included in the case series? Q4: Did the case series have consecutive inclusion of participants? Q5: Did the case series have complete inclusion of participants? Q6: Was there clear reporting of the demographics of the participants in the study? Q7: Was there clear reporting of clinical information of the participants? Q8: Were the outcomes or follow-up results of cases clearly reported? Q9: Was there clear reporting of the presenting site(s)/clinic(s) demographic information? Q10: Was statistical analysis appropriate?

### Meta-analysis results

#### Rates of technical success and clinical response

**FIGURE 2:**
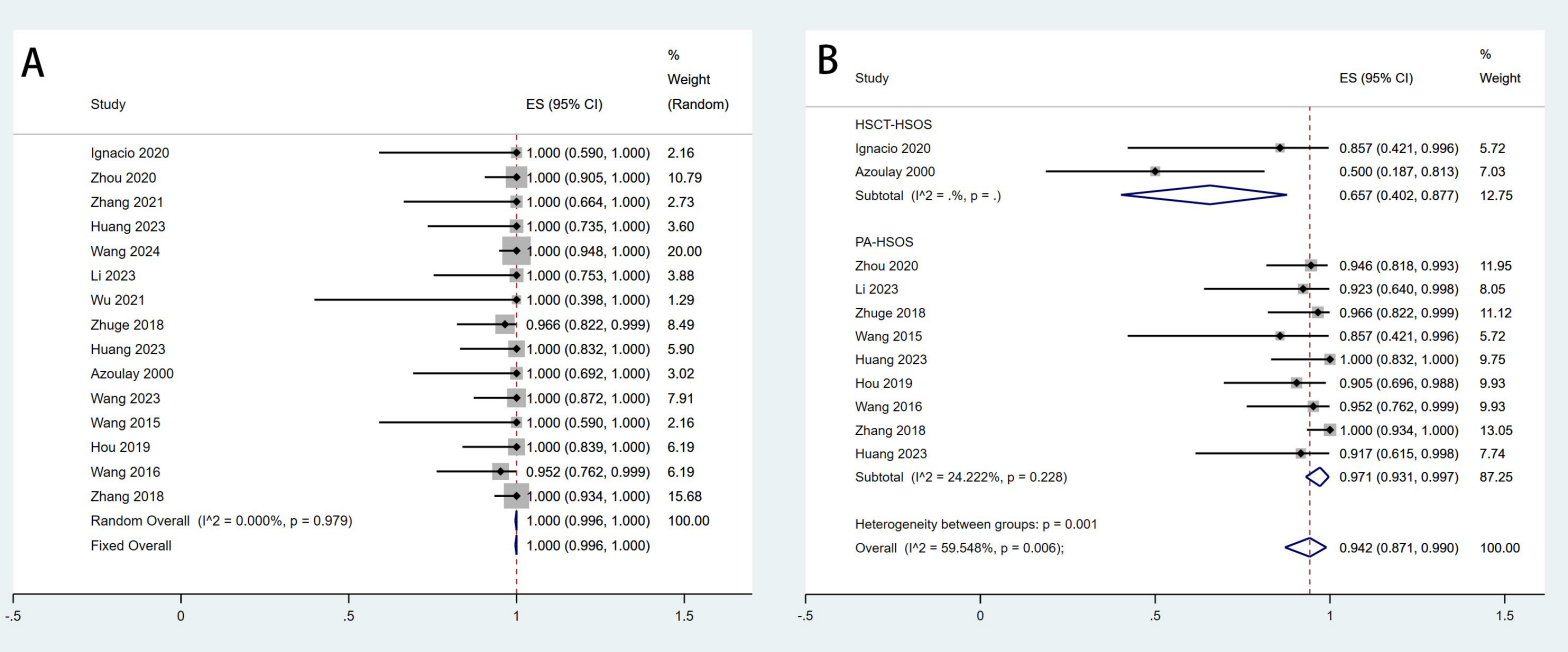
Meta-analysis of the rates of technical success **(A)** and clinical response **(B)** after TIPS in patients with HSOS.

The establishment of a shunt between the hepatic vein and an intrahepatic branch of the portal vein is considered a technical success for TIPS (17). A total of fifteen studies involving 340 patients reported the technical success rate, with a pooled success rate of 100% (95% CI: 99.6–100%, *I*^2^ = 0%). Clinical response was defined as a marked alleviation or resolution of clinical symptoms within 2 weeks of medical treatment, accompanied by improvements in laboratory and imaging findings (19). A total of eleven studies involving 231 patients reported the clinical remission rate, with a pooled remission rate of 94.2% (95% CI: 87.1–99.0%, *I*^2^ = 59.5%). Additionally, subgroup analyses were performed based on etiology to explore potential factors contributing to the significant heterogeneity in clinical remission rates. The results revealed significant overall heterogeneity in effect size. However, after subgroup analysis, within-group heterogeneity was no longer significant, suggesting that the subgrouping factor was likely a major contributor to the observed heterogeneity (Figure 2).

#### Change in portal pressure gradient and portal venous pressure

**FIGURE 3:**
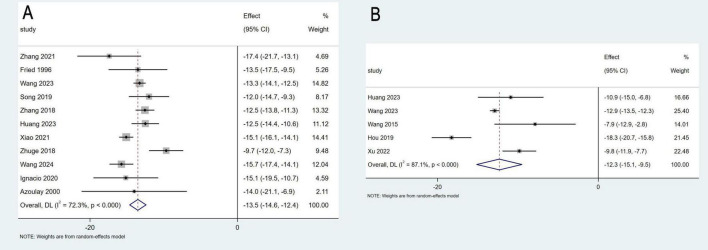
Meta-analysis of the change in portal pressure gradient **(A)**, and portal venous pressure **(B)**.

**FIGURE 4:**
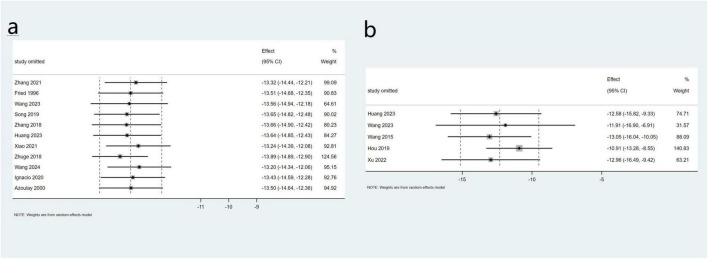
Sensitivity analysis of PPG **(a)** and PVP **(b)** in the total study.

Eleven studies involving 355 patients reported data on changes in PPG, while five studies with a total of 82 patients provided data on PVP. The pooled mean change in PPG was −13.5 mmHg (95% CI: −14.6 to −12.4 mmHg, *I*^2^ = 72.3%), and the pooled mean change in PVP was −12.3 mmHg (95% CI: −15.1 to −9.15 mmHg, *I*^2^ = 87.1%) (Figure 3). Sensitivity analyses were performed by systematically excluding individual studies from the pooled results, and these analyses demonstrated that such exclusions did not materially affect the overall findings (Figure 4).

Potential publication bias among the included studies was assessed using Begg’s and Egger’s tests. For the pooled PPG results, Egger’s test (*P* = 0.828) and Begg’s test (*P* = 0.755) indicated no significant publication bias. Similarly, for the pooled portal PVP results, Egger’s test (*P* = 0.809) and Begg’s test (*P* = 0.806) also showed no evidence of publication bias. A meta-regression analysis was performed for the pooled PPG results, incorporating etiology as a covariate. The *p*-value for etiology was 0.674 (*P* > 0.05), suggesting that etiology was not a significant contributor to heterogeneity. Due to the limited number of studies reporting PVP data, a meta-regression analysis for PVP was not conducted.

#### Rates of survival and hepatic encephalopathy

**FIGURE 5:**
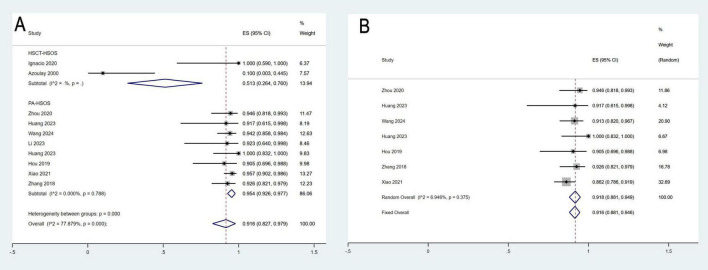
Meta-analysis of the survival rates of 3-month **(A)** and 1-year **(B)**.

**FIGURE 6:**
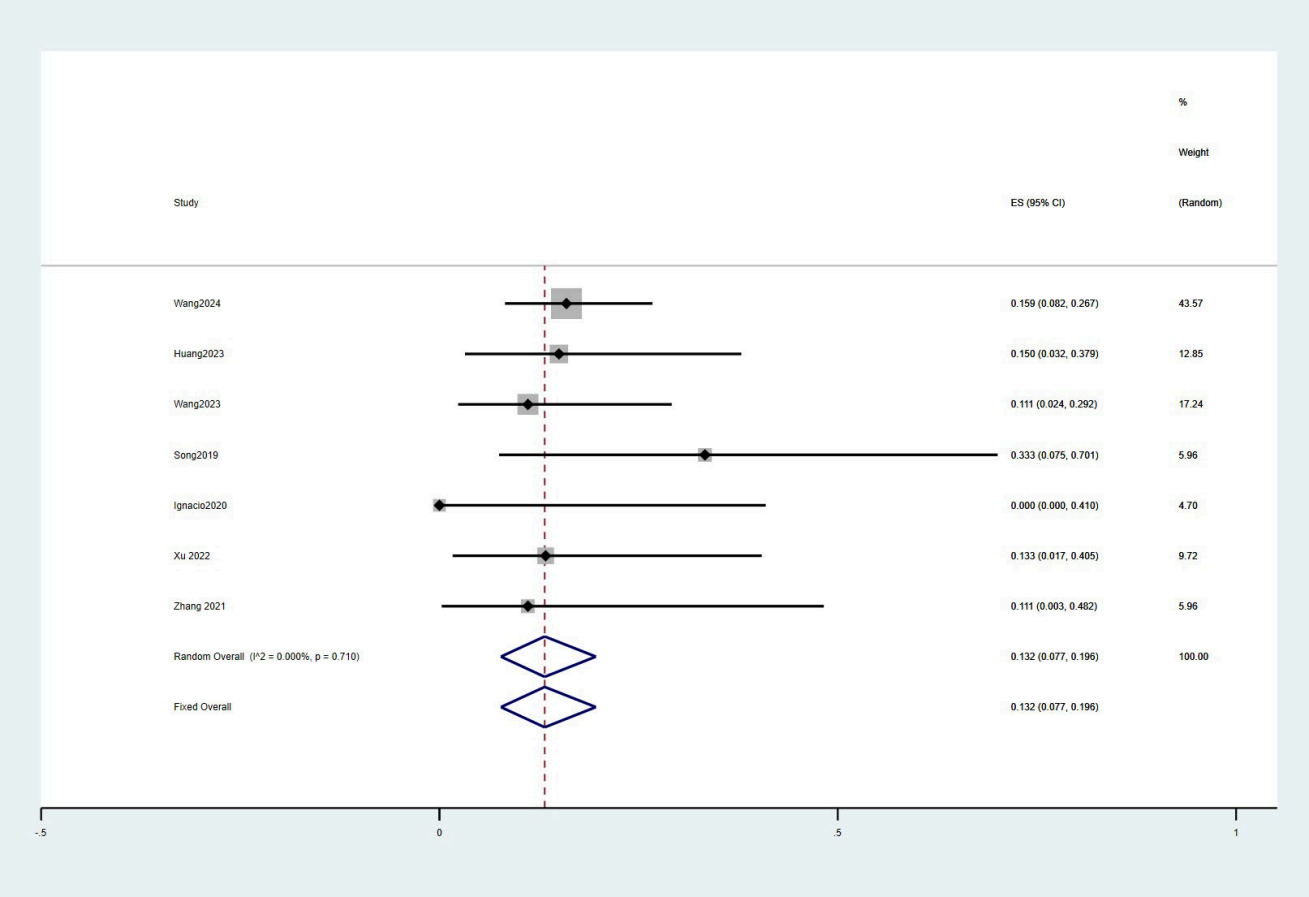
Meta-analysis of the incidence of HE.

A total of ten studies involving 359 patients reported 3-month survival rates, with a pooled survival rate of 91.6% (95% CI: 82.7–97.9%, *I*^2^ = 77.9%). Seven studies with 329 patients provided data on 1-year survival rates, resulting in a pooled survival rate of 91.6% (95% CI: 88.1–94.6%, *I*^2^ = 6.9%) (Figure 5). Additionally, eight studies involving 225 patients reported the incidence of HE, with a pooled incidence rate of 13.2% (95% CI: 7.7–19.6%, *I*^2^ = 0%) (Figure 6).

To explore potential factors contributing to heterogeneity in the 3-month survival rate, a subgroup analysis based on etiology was conducted. As shown in the results, although overall heterogeneity in the effect size was significant, subgroup analyses revealed no significant heterogeneity within the subgroups. These findings suggest that etiology may be a significant source of heterogeneity.

#### Subgroup analysis by etiology

To address the observed heterogeneity in treatment outcomes, we conducted a subgroup analysis based on etiological characteristics (PA-HSOS vs. HSCT-HSOS). The results showed that the pooled clinical response rate for PA-HSOS patients was 97.1%, whereas it was only 65.7% for HSCT-HSOS patients. Similarly, the pooled 3-month survival rate was 95.4% in the PA-HSOS subgroup, compared to just 51.3% in the HSCT-HSOS subgroup. These findings are illustrated in Figures 2B, 5A and are systematically summarized in Table 3. These results suggest a significant association between etiology and treatment efficacy, indicating that etiology may be a key contributor to the overall heterogeneity observed in the analysis.

**TABLE 3:** Summary of pooled outcomes by etiology.

Outcome	PA-HSOS (*n* = 10 studies)	HSCT-HSOS (*n* = 2 studies)
Clinical response rate	205/214 = 95.8%	11/17 = 64.7%
3-month survival rate	323/342 = 94.4%	8/17 = 47.1%

PA-HSOS, pyrrolizidine alkaloid-induced hepatic sinusoidal obstruction syndrome; HSCT-HSOS, hematopoietic stem cell transplantation-induced hepatic sinusoidal obstruction syndrome.

## Discussion

Portal hypertension and liver failure are the key pathological mechanisms driving disease progression in patients with HSOS. Common clinical manifestations include abdominal distension, ascites, and hepatomegaly (1). Current treatment strategies primarily focus on symptomatic management. However, in severe cases, symptomatic and supportive treatment alone is often insufficient to significantly reduce mortality. (6, 31, 32). For critically ill patients, liver transplantation can also be considered. However, it has inherent limitations, including organ shortages, surgical risks, and high costs (33). For patients who do not respond to symptomatic treatment and are awaiting liver transplantation, TIPS may serve as a viable alternative. The results of this study show that TIPS treatment led to a mean reduction in PPG of 13.5 mmHg and a mean reduction in PVP of 12.3 mmHg. This significant decrease in pressure effectively alleviated clinical symptoms associated with portal hypertension, such as abdominal distension, ascites, and hepatomegaly. Moreover, the pooled 3-month and 1-year survival rates reached 91.6%, suggesting that TIPS may improve the survival prognosis in these patients.

In patients with hematopoietic stem cell transplantation-induced hepatic sinusoidal obstruction syndrome (HSCT-HSOS), the only drug currently approved is defibrotide, which has a 100-day survival rate of approximately 50% (34, 35). However, the efficacy of TIPS in patients who are unresponsive to defibrotide remains controversial. Early studies in the 2000s suggested that TIPS did not significantly improve survival in these patients, with a 3-month survival rate of only 10% (36). Nevertheless, more recent studies have indicated that TIPS may improve survival and provide additional time for liver transplantation, reflecting advancements in interventional techniques and supportive care. Currently, research on the use of TIPS for HSCT-HSOS remains limited and requires further validation through large-scale randomized controlled trials.

In PA-HSOS patients, since defibrotide has not been approved for use in China, no studies have evaluated its efficacy in this population (20). In clinical practice, a stepwise approach of initial anticoagulation followed by TIPS is widely adopted. Existing evidence suggests that in mild cases, symptomatic or anticoagulation therapy may achieve comparable efficacy to TIPS; however, in severe cases, TIPS tends to show better clinical benefit (20). In this study, TIPS treatment for PA-HSOS patients demonstrated good safety and efficacy, with a 3-month survival rate and technical success rate reaching 95% and 100%, respectively. These results indicate that TIPS is both feasible and potentially valuable for broader application in PA-HSOS patients.

The efficacy of TIPS differs between PA-HSOS and HSCT-HSOS patients, primarily because mortality in HSCT-HSOS is often linked to multi-organ failure, sepsis, and hemorrhage, typically resulting from underlying hematological disorders. In contrast, PA-HSOS patients usually do not have severe hematological diseases, presenting with milder conditions and a slower progression of the disease (13).

In addition, regarding concerns about TIPS-related complications, the incidence of HE in this study was 13.2%, which is comparable to the incidence observed in other end-stage liver disease patients undergoing TIPS treatment, with no significant increase observed (37).

This study is the first systematic review to evaluate the efficacy of TIPS in the treatment of HSOS patients using quantitative meta-analysis. Like any meta-analysis, this study has several limitations. First, all included studies were retrospective observational in nature, which limits causal inference and introduces selection bias. TIPS is typically reserved for patients with more severe disease, leading to potential confounding by indication. Second, only single-arm studies were included, without direct comparisons to other treatments such as anticoagulation or defibrotide. Third, although our etiology-based subgroup analysis partially explained the clinical heterogeneity in treatment response (*I*^2^ = 59.5%), other factors—such as age, baseline liver function, coagulation status, and portal vein velocity—may also contribute. However, most included studies did not consistently report these variables, limiting further subgroup or meta-regression analyses. Lastly, most studies focused on PA-HSOS, while data on HSCT-HSOS were limited; thus, our findings are more generalizable to the PA-HSOS population. Future prospective, multicenter studies with standardized reporting are needed to validate these results and clarify the role of TIPS in current treatment algorithms.

## Conclusion

Overall, TIPS treatment for HSOS is both feasible and effective, significantly reducing portal pressure and lowering the risk of major complications. Therefore, TIPS should be considered a viable treatment option for HSOS patients, particularly for those with PA-HSOS. However, due to the current lack of randomized controlled trials, further studies are needed to assess its long-term safety and efficacy.

## Data Availability

The original contributions presented in this study are included in this article/supplementary material, further inquiries can be directed to the corresponding author.
